# Barriers and Constraints in Scientific Manuscript Preparation Among Nephrologists: Insights From India

**DOI:** 10.1155/ijne/9008616

**Published:** 2025-02-23

**Authors:** Mythri Shankar, Anaghashree Udayashankar, Sowrabha Rajanna, Urmila Anandh, Arpita Ray Chaudhury

**Affiliations:** ^1^Department of Nephrology, Institute of Nephrourology, Bengaluru, Karnataka, India; ^2^Department of Nephrology, Medicover Hospital, Bengaluru, Karnataka, India; ^3^Department of Nephrology, JSS Medical College, JSS Academy of Higher Education and Research, Mysore, Karnataka, India; ^4^Department of Nephrology, Amrita Institute of Medical Sciences, Faridabad, Delhi NCR, India; ^5^Department of Nephrology, NBMCH, Siliguri, West Bengal, India

**Keywords:** challenges, limitations, scientific paper writing

## Abstract

**Introduction:** Medical research shapes public health actions, emphasising the need for greater investments in health. Despite a surge in scientific publications, disparities exist in authorship from low-income countries and among female researchers. Addressing these gaps is vital for studying real-world health outcomes and promoting universal healthcare delivery.

**Methods:** A descriptive quantitative study using an online questionnaire to gather data from Indian nephrologists and nephrology fellows was conducted by members of Women in Nephrology, India, from September 2023 to December 2023. The survey collected data on demographics, publication experience and challenges in scientific paper writing. Statistical analyses were performed using SPSS Version 25.0, with significance at *p* < 0.05.

**Results:** The survey included 156 participants, with a mean age of 35.55 ± 8.91 years. The majority were males (55.8%) and practicing nephrologists (69.9%). Most respondents practiced in medical institutions (45.5%) and metropolitan cities (60.3%), with an average practice duration of 12.29 ± 9.12 years. Only 44.9% published their thesis work, and 52.6% preferred writing case reports. Key challenges included time constraints (82.1%), funding (67.9%), limited access to research articles (65.4%), lack of statistical analysis knowledge (64.7%) and limited access to research software (60.2%). Younger nephrologists faced more funding (68.9%) and knowledge-related barriers (74.4%).

**Discussion:** Multiple challenges exist in scientific paper writing among Indian nephrologists, emphasising the need for targeted interventions. Funding for research, burnout and article processing charges are significant barriers. Addressing these challenges is crucial for enhancing research output and improving healthcare outcomes in resource-limited countries.

**Summary**

• The study highlights the significant barriers faced by Indian nephrologists in scientific publishing.
• The research was conducted via an online questionnaire involving 156 participants, involving practicing nephrologists and nephrology fellows, to understand the challenges they encounter.
• Key obstacles include time constraints, funding, limited access to research articles and software and a lack of knowledge in statistical analysis.
• Younger nephrologists face more issues with funding and resources, while women are now publishing more frequently despite additional nonprofessional responsibilities.
• Addressing these challenges is essential for improving scientific output and healthcare in resource-constrained environments.
• The study calls for targeted interventions, particularly for authors from low-income countries, to enhance research opportunities and bridge gender and geographic disparities in scientific publications.

## 1. Introduction

Research drives science, and medical sciences are no exception. Evidence-based medicine enhances healthcare decisions [1]. Medical research shapes public health dialogue, emphasising the need for greater and targeted health investments. Scientific publications, as written documents of research findings, play a crucial role in disseminating outcomes within the scientific community. Furthermore, the publication of research is considered evidence of an institution's research capacity and significantly influences university rankings and future funding prospects [2, 3].

In recent years, there has been a surge in scientific publications across all fields, including nephrology. This increase is attributable to new molecular discoveries, a rise in clinical trials and enhanced accessibility to publications through the efforts of various scientific societies and prominent journals [4, 5].

Despite the overall increase in research publications, there is a notable lack of representation from low- and middle-income countries (LMICs) and women in first or senior authorship roles. A study by Subhash Chander et al. reported that the median proportion of women in first and senior authorship positions across eleven prominent journals was 20.8% (interquartile range: 13.3%–25.3%) and 9.4% (8.6%–12.4%), respectively [6].

Promoting an inclusive and diverse research community is crucial. Bridging these gaps is imperative to study real-world health outcomes and promote universal healthcare delivery.

This study aims to investigate the obstacles perceived by nephrologists in writing scientific papers, as no data on this subject have been available to date. Identifying these obstacles and aids is essential, as only through this understanding can customised assistance be developed to enhance the writing process and promote a diverse research community.

## 2. Methodology

A questionnaire-based survey was developed to gather data and insights regarding challenges and limitations in scientific paper writing from an Indian perspective.

### 2.1. Study Design

The study utilised a descriptive quantitative research design and was conducted collaboratively by members of Women in Nephrology, India (WIN-India) [7].

WIN-India is an organisation in India consisting of over 400 women nephrologists in the country. WIN-India was established in August 2021 to provide mentorship and a support system to the Indian nephrology community by promoting research activities, conducting academic programmes, networking, advocacy and leadership development.

### 2.2. Study Population

The study population consisted of consultant nephrologists and nephrology fellows, across India between September 2023 and December 2023.

### 2.3. Sampling Strategy and Data Collection

The sampling strategy employed was a convenient sampling method. The communication details of the respondents were obtained from the social network nephrology communities and groups from numerous institutions and different regions. The questionnaire was then circulated using an online survey program that allows the respondents to answer the questions and send us their responses by electronic mail. The link to this online survey was sent by electronic mail and also through social media platforms such as WhatsApp and Twitter among the study population. The respondents were requested to complete the survey in 1 month. A reminder email was sent at 15 days. Participation was voluntary and responses were anonymous. No identifying data were collected.

The survey was conducted in accordance with the Declaration of Helsinki. Electronic consent was obtained from all participants and responses were discreetly recorded. The anonymity of the subjects was safeguarded. Formal ethical approval was considered not necessary for this voluntary online survey of healthcare professionals, which used anonymised data.

### 2.4. Research Instrument

A cross-sectional observational questionnaire-based survey was utilised to collect the data from study subjects. This questionnaire was developed based on previous studies exploring the barriers of scientific writing [3, 8–11].

The initial part of the survey reviewed 10 items comprising the participants' age, gender, place of practice, duration of nephrology practice, number of publications, number of poster/podium presentations and type of research study preferred by the participant.

The second section of the survey evaluated the key challenges faced during paper writing. It was assessed using 20 items using a 5-step Likert scale ranging from strongly disagree to strongly agree.

The third section of the survey appraised the significant limitations identified during paper writing using 9 critical areas, using a 5-step Likert scale ranging from strongly disagree to strongly agree. The survey also gathered data regarding the number of rejections and revisions faced during submission of an article.

*Challenges* refer to obstacles or difficulties that nephrologists face during the process of conducting research and writing papers. These are often external or procedural hurdles that can affect the efficiency and effectiveness of the research process. These challenges are generally operational issues that can potentially be managed or mitigated through training, better resource allocation or policy changes.

*Limitations*, on the other hand, refer to intrinsic constraints within the study itself that may affect the validity or generalisability of the findings. These are typically factors that are not within the direct control of the researchers and can lead to biases or weaknesses in the study design or execution.

### 2.5. Analysis of Challenges and Limitations

These two categories are analysed through different lenses:

• Challenges are analysed to identify systemic issues in the research process that could be addressed through changes in practice, policy or resources.
• Limitations are used to caution readers about the potential biases or interpretative issues that might affect the trustworthiness or applicability of the research findings.

### 2.6. Statistical Analysis

Descriptive and inferential statistical analysis has been carried out in the present study.

Results of continuous measurements are presented as mean ± SD (min–max), and results of categorical measurements are presented as number (%). Significance is assessed at 5% level of significance.

The following assumptions on data are made: Assumption 1: Dependent variables are normally distributed. Assumption 2: Samples drawn from the population are random, and cases of the samples are independent.

The chi-square test has been used to find the level of significance of study parameters on categorical scale between two or more groups in nonparametric setting for qualitative data analysis.

Fisher's exact test has been used when cell samples are very small.

The study outlines the operationalisation of statistical significance using specific terms and corresponding *p* values, which help clarify the degree of evidence against the null hypothesis. Here is a breakdown of how these terms are defined in the study:

1. Suggestive of significance (*p* value: 0.05 ≤ *p*  < 0.10):
    This range indicates that the findings are close to being statistically significant but do not meet the traditional threshold of 0.05. It suggests there is some evidence against the null hypothesis, but it is not strong enough to be considered conclusive.
2. Moderately significant (*p* value: 0.01 ≤ *p* < 0.05):
    Results within this range are considered statistically significant at a moderate level. This means that there is less than 5% probability that the observed results are due to chance, providing stronger evidence against the null hypothesis.
3. Strongly significant (*p* value: *p* ≤ 0.01):
    Findings with *p* values at or below 0.01 are considered to have strong statistical significance. This suggests a very low probability (less than 1%) that the results occurred by chance, indicating very strong evidence against the null hypothesis.

Statistical software: The statistical software types namely SPSS 22.0 and R environment Version 3.2.2 were used for the analysis of the data. Microsoft Word and Excel have been used to generate graphs and tables.

## 3. Results

We reached out to 534 nephrologists and nephrology fellows via WhatsApp and email, sharing the survey forms. Of these, 156 participants responded to the survey and completed it. The response rate for the survey was approximately 29.21%. The number of responses per question was 156 (100%). This survey, which included 156 practising nephrologists and fellows, revealed some key demographics. The majority fell within the 31–40 years age range (53.8%), with a mean age of 35.55 ± 8.91 years. Males made up the majority at 55.8%. 69.9% of practising nephrologists and 30.12% of nephrology fellows participated in the study. Most respondents (45.5%) practised in medical colleges/institutions, with only a small percentage (0.6%) focussing entirely on research. Most were from metropolitan cities (60.3%), and the average duration of practice in nephrology was 12.29 ± 9.12 years (Table 1). *Thesis is a required component of nephrology training in India.* Only 44.9% of the respondents published their thesis work in nephrology. 52.6% found case reports easy to write and publish, followed by retrospective observational studies (17.9%). Case–control studies and randomised control studies were the least preferred types of research. 38.5% had presented their research work as podium or poster presentations 2 to 5 times and 28.5% more than five times. Out of these presentations, the majority (72.4%) published only one research article (Table 2).

The top 5 challenges in scientific paper writing faced by the respondents were as follows (Table 3):

1. Time constraints (82.1%)
2. Funding for research (67.9%)
3. Limited access to research articles due to paywall (65.4%)
4. Lack of knowledge on statistical analysis (64.7%)
5. Limited access to software for research analysis (60.2%)

39.7% of the respondents shared that their articles were rejected atleast once before being accepted by another journal. 37.8% said they had to revise the article 2 to 5 times before it was accepted.

The top 5 limitations of the respondents for scientific paper writing were the following concerns (Table 4):

1. ‘Article processing charges (APCs) for journals is a waste of money' (70.5%)
2. ‘I have financial constraints' (55.8%)
3. ‘Demand for increased number of revisions by the editors puts me off' (53.2%)
4. ‘I doubt if my results will have any major real-world impact' (45.6%)
5. ‘I'm afraid my scientific paper may not receive good number of citations despite the hardwork' (43%)

The study also delved into the effect of age on scientific paper writing, the results of which are depicted in Supporting Table 1. It was found that older nephrologists (> 50 years) had significantly higher numbers of poster/podium presentations (81.8% vs. 24.82%, *p* < 0.001) and publications (12.8% vs. 9.65%, *p* < 0.001) compared to younger groups.

Younger nephrologists, on the other hand, found funding for research (68.96% vs. 54.6%, *p*=0.01), lack of knowledge in statistics (74.4% vs. 63.7%, *p*=0.01), limited access to software for research analysis (65.1% vs. 54.6%, *p*=0.04) and difficulty in literature review (51.1% vs. 9.1%, *p*=0.007) as significant challenges in scientific paper writing compared to the older age group. Burnout was significantly higher among nephrologists between 20 and 30 years compared to those beyond 50 years (74.4% vs. 27.3%, *p*=0.002). Clearly, the obstacles faced by the younger age group was higher than the older age group. This could be attributed to the longer career length of senior nephrologists (Supporting Table 1).

The challenges of paper writing with respect to the gender of the doctors were also studied; the results are shown in Supporting Table 2. Women doctors had significantly more podium/poster presentations compared to men (40.6% in women vs. 19.5% in men, *p*=0.008); also they published more compared to men (18.8% in women vs. 8% in men, *p*=0.036). Men found difficulty in framing research questions as a major challenge (40.1% in women vs. 48.3% in men, *p*=0.02), while women expressed that lack of research interest was their major challenge (36.7% in women vs. 24.6% in men, *p*=0.018) (Supporting Table 2).

## 4. Discussion

There are multiple challenges in healthcare delivery in the developing world. Enhancing research is one of the most effective ways of improving the status of health care in this resource constrained nations [12, 13]. Despite this, research continues to be a low priority in physicians in these countries as there are many barriers to research and scientific publishing. In a study from India, more than half of medical college faculty had not had any publications in a decade, starting in 2005 [14]. This survey tries to highlight some of the areas of concern.

One of the most significant concerns cited by 67.9% of the respondents in this survey was funding. The nonavailability of sufficient financial resources as greatest barrier to research activity has also been emphasised by Kabirpanthi, Gupta and Chavan [15]. Not only the financial support for high quality research is lacking (especially in noncommunicable diseases which are the major risk factors for chronic kidney disease) [16], but also funding for publications is becoming a major concern in the ‘open-access' (OA) era. This in-turn creates more opportunity for ‘vanity presses' [17–19] and also makes publishing extremely difficult for authors from India.

Astronomical APCs prohibit authors from the global south (*LMICsin Africa, Latin America and parts of Asia)* from publishing in prestigious journals with high impact factor [20, 21]. Many journals partially waive off APCs, still many LMICs remain ineligible to apply for these waivers [22]. Authors from India cannot apply for reduced APCs as they belong to a large economy, even though the per capita income is comparatively low. The unfortunate consequence of APCs is that many authors cannot afford the amount [23] and fall in the trap of publishing their work in predatory journals [24].

The observation that majority (70.5%) of the survey respondents felt that APCs were a huge expenditure underscores a critical barrier for researchers from LMICs. These charges disproportionately impact researchers with limited funding, paradoxically hindering the very goal of broad dissemination that OA aims to achieve. A deeper exploration into this issue includes the economic impact on researchers, the role of institutional and governmental support in subsidising these costs and innovative publishing models that circumvent the need for APCs.

Highlighting the need for policy changes and incorporation of collaborative funding models at national and international levels could offer viable solutions. The collaborative funding models pool funds from national or regional organisations and philanthropists to fund research projects. It can alleviate the challenges and ensure a more strategic approach to address the resource gaps and deficits in funding. It also expedites the research process and leads to novel discoveries [25].

On the other hand, the government could allocate more funds for research and development in the national budget providing scholarships and fellowships for aspiring researchers. The medical universities must seek to form collaborations with other institutions for monetary support. They could enter into membership agreements with certain publishers to minimise the subscription and publication fees for journals to make research more accessible for physicians from LMICs.

Such an expanded discussion would not only address the financial challenges but also propose actionable strategies, advocating for a more equitable scientific publishing landscape that enhances the visibility and impact of global research.

Another notable finding is that fellows in India do not publish their thesis. This is also similar to data from the first world, where the majority of the scientific presentations in meetings as abstracts are not published as original articles [26]. In Nigeria, lack of time because of clinical responsibilities was a major impediment [27]. In China, the students felt they needed inspiring mentors to guide them [28]. In the Arab states, the lack of equipment and infrastructure plays an important role [29, 30]. These barriers in various forms are also expressed in this survey.

Another important finding that came out of the survey is the fact that young women nephrologists are increasingly moving towards publishing their work. This is a very welcome trend as this is in contradiction to literature from rest of the world. A review of literature reveals that women scientists publish much less than their male counterparts do. Women have greater nonprofessional responsibilities, work in challenging work environments and often need help to break the glass ceiling. This is true across the spectrum of publications in science, not just in medicine. Nonprofessional responsibilities often attributed to women vary widely by culture, society and individual choices. Traditionally, these responsibilities have included a range of domestic and caregiving roles. Some common areas where women have often played a significant role are household management, childcare, elderly care, emotional support, community engagement and children's education, though it is important to recognise that these roles are changing and that there is increasing recognition of the need for shared responsibilities between all genders [30, 31].

Women are less likely to be named as authors in a manuscript; their work less recognised and less likely to be credited as their work is not known [32]. They also have less patents compared to their male peers [33]. The most celebrated instance of women overlooked when it was due is the case of Ms Rosalind Franklin who was one of the scientists involved in elucidating the structure of the DNA. She was wrongfully denied her role, and it was only after her death the truth was known [34]. Women as first authors of manuscripts in high impact journals is also quite rare [35]. One of the reasons could be these high impact journals preferentially publish large randomised trials and women get less funding for such trials [36–38]. However, this trend is changing [39]. Hopefully this will manifest as more women clinicians will take up scientific writing and publish more often as this survey indicates. In India, women currently constitute 60% of nephrology fellows, indicating a significant shift in trends within the field [40].

Academic burnout is being increasingly encountered in Asian countries, across various specialties during residency programs and in professional practice. The negative impact of this condition overwhelms the ability of students and practitioners to cope with daily challenges and leads to emotional exhaustion. The balance between work–life expectations and personal accomplishments is threatened, thus compromising productivity at physical, mental and social levels [41].

Time management issues are the most frequently mentioned physical barriers. Academicians cited several reasons for these problems, including the inability to find time to write, procrastination, poor daily and long-term planning, inability to complete tasks during the day and sacrificing sleep.

This survey identifies several key challenges that frequently necessitate revisions in the scientific paper writing process. Faulty or unclear methodology is a primary issue, often leading to extensive revisions when reviewers request clarification or additional experiments. Additionally, many respondents reported a significant lack of knowledge in statistical analysis, resulting in errors and inappropriate use of statistics that require corrections to validate research findings. Language barriers also present a notable challenge, especially for nonnative English speakers, compelling revisions to enhance manuscript readability and coherence. Overall, these revisions are common across methodology, statistical analysis and language, with the extent of their impact varying based on the researcher's background and the specific demands of the research.

To mitigate these challenges, the following measures are suggested:

• Develop a structured writing schedule and adhere to it. Break the paper into smaller, manageable sections and set deadlines for each.
• Focus on priority sections or stages of research paper first.
• Use tools like Gantt charts to visualise and manage tasks efficiently.
• If possible, delegate research or writing tasks to other team members or collaborators to distribute the workload and accelerate the writing process.
• Explore a variety of funding sources including grants, fellowships and sponsorships from scientific societies, government bodies and private foundations.
• Carefully plan and manage budget to prioritise essential resources like OA publishing fees or necessary software.
• For smaller projects or preliminary studies, consider crowdfunding platforms geared toward science and research.
• Utilise institutional accesses such as those provided by universities or research institutes. For articles not available in library, use interlibrary loan services. The recent proposal by the Government of India to purchase a nationwide subscription to 13,000 journals, under ‘One Nation-One Subscription' scheme scheduled to begin on January 1, 2025, is a welcoming step [42].
• Focus on using and citing OA resources that are freely available. Request copies directly from authors, who are often willing to share PDFs of their published papers. Understand and communicate the value of OA—broader dissemination and increased accessibility can lead to a greater impact, which may justify the expense.
• Select journals that balance the impact with reasonable processing charges or offer waivers and discounts based on the researcher's country of origin or financial situation.
• Seek support from your institution or research grants specifically allocated for publication fees. Include publication costs in grant applications and budget planning from the onset of the research project.
• Investigate journals that offer waivers or discounts for researchers from low-income countries or those with limited funding.
• Consider collaborative projects where costs can be shared among multiple institutions or researchers.
• View revisions as a normal part of the scientific publishing process meant to refine and enhance your work's quality. Engage colleagues to review your paper before submission to minimise the extent of revisions required by journal editors. Utilise professional editing services or mentors who can help streamline your manuscript in accordance with high editorial standards before submission.

To address the challenges in the evaluation process, several strategies are proposed such as reviewing the guidelines and scope of the journals, enhancing the text with images for better clarity, seeking advice from authors who have submitted to similar journals, preassessing the manuscript from a reviewer's perspective, examining recent publications in the target journal and reaching out to the editor [43].

Challenges regarding knowledge of statistics and use of statistical softwares can be circumvented by conducting regular courses in scientific writing and incorporating the curriculum during residency or fellowship courses [44].

By systematically addressing each of these challenges and limitations, researchers can efficiently contribute to scientific writing process and enhance their productivity and the quality of their work.

The potential limitations of this study include the use of a nonvalidated survey tool. The data collected were based on self-reported measures, which can introduce biases such as social desirability bias or recall bias. This may affect the accuracy of the information regarding challenges and limitations experienced by the participants. Given the sensitive nature of some topics, such as financial constraints and personal perceptions of the impact of research, there might be underreporting of these issues. Participants may be reluctant to fully disclose their struggles in a survey, even if it is anonymous.

On the other hand, the fact that only 156 out of 534 participants responded to the survey shows that people are frequently hesitant to spend time answering the survey-based questionnaires. This is another limitation of survey-based research. Apart from being circulated through regional platforms like email or social media, the survey could have been officially distributed by the institutions themselves, which could have boosted the response rate.

## 5. Conclusion

The study highlights the multiple challenges in scientific paper writing among Indian nephrologists, emphasising the need for targeted interventions. Funding remains a significant barrier, exacerbated by high article processing charges, which limit publication opportunities for authors from low-income countries. Young women nephrologists are encouragingly publishing their work, indicating a positive trend towards gender parity in scientific contributions. Addressing these challenges is crucial for enhancing research output and improving healthcare outcomes in resource-constrained settings.

1. Bachelor of Medicine, Bachelor of Surgery (MBBS): This is the primary medical degree awarded by medical schools in India and many Commonwealth countries. It is equivalent to the Doctor of Medicine (MD) in the United States and is the foundational qualification required to practice medicine.
2. Doctor of Medicine (MD): In the context of Indian medical education, an MD is a postgraduate degree awarded after 3 years of study in a particular specialty. It is not to be confused with the primary medical qualification in countries like the United States, where the MD is the basic medical degree.
3. Diplomate of National Board (DNB): The DNB is an equivalent qualification to the MD and is awarded after completing postgraduate training in hospitals recognised by the National Board of Examinations. It involves passing an exam that assesses the candidate's knowledge and skills in their chosen specialty.
4. Membership of the Royal Colleges of Physicians (MRCP): This is a postgraduate medical diploma in the United Kingdom. The MRCP qualification is recognised internationally and indicates that the holder has passed rigorous assessments and can practice as a consultant physician in the Commonwealth countries.
5. Superspecialisation: This generally refers to further specialised training taken after completing an MD or DNB, often leading to qualifications such as Doctorate of Medicine (DM) in a specific medical field such as cardiology, neurology or nephrology, or Master of Surgery (MCh) for surgical subspecialties.
6. Senior Residents or Doctorate of National Board (DRNB)/DM Trainees: These are doctors who are undergoing specialised training in their chosen field. They are more experienced than general residents and are usually in the final stages of their postgraduate medical education.

## Figures and Tables

**Table 1:** Baseline demographics of study participants.

Age in years	No. of doctors (*N* = 156)	%	Mean age ± SD
21–30	43	27.6	35.55 ± 8.91
31–40	84	53.8	
41–50	18	11.5	
51–60	6	3.8	
61–70	5	3.2	
Total	156	100.0	

**Gender**	**No. of doctors**	**%**	

Female	69	44.2	
Male	87	55.8	

**Qualifications**⁣^∗^	**No. of doctors**	**%**	

Superspecialisation	61	39.1	
MD	46	29.5	
DNB (nephrology)	46	29.5	
MRCP	46	29.5	
MBBS	31	19.9	
Senior Residents or DRNB/DM Nephrology Trainees	20	12.8	

**What is your predominant current area of practice?**	**No. of doctors**	**%**	

Medical college	71	45.5	
Corporate	61	39.1	
Private practice	23	14.7	
Research	1	0.6	

**Place of practice**	**No. of doctors**	**%**	

Metropolitan city	94	60.3	
Smart city⁣^∗∗^	35	22.4	
Town	23	14.7	
Village	4	2.6	

**Total years of practice (from the time of MBBS completion)?**	**No. of doctors**	**%**	**Mean ± SD**

1–10	83	53.2	12.29 ± 9.12
11–20	47	30.1	
21–30	19	12.2	
> 30	7	4.5	

⁣^∗^The qualifications of the participants are relevant as they provide insight into the level of expertise and experience of the respondents, which can influence their perceptions of the challenges they face in scientific writing.

⁣^∗∗^The term ‘smart city' refers to urban areas that are specifically designed to be highly efficient, technologically integrated and sustainable through the use of digital technology.

**Table 2:** Information on publications.

Did you publish your thesis?	No. of doctors	%
No	74	47.4
Yes	70	44.9
Not applicable	12	7.7

**What do you find easy to do and write?**	**No. of doctors**	**%**

Case reports	82	52.6
Retrospective observational	28	17.9
Cohort-prospective observational study	19	12.2
Case series	16	10.3
Randomised controlled trials	7	4.5
Case–control studies	4	2.6

**How many podium/poster presentations have you done so far?**	**No. of doctors**	**%**

Less than 2	51	32.7
2–5	60	38.5
More than 5	45	28.8

**How many podium/poster presentations got converted into paper publications?**	**No. of doctors**	**%**

Less than 2	113	72.4
2–5	23	14.7
More than 5	20	12.8

**On an average, how many times were your papers rejected before being accepted?**	**No. of doctors**	**%**

Less than 2	62	39.7
2–5	46	29.5
More than 5	3	1.9
Not applicable	45	28.8

**On an average, after how many revisions were your papers accepted?**	**No. of doctors**	**%**

Less than 2	54	34.6
2–5	59	37.8
More than 5	1	0.6
Not applicable	42	26.9

**Table 3:** Challenges faced in scientific paper writing.

What are the challenges that you face in scientific paper writing? (*n* = 156)	Strongly disagree	Disagree	Neither agree/nor disagree	Agree	Strongly agree
Difficulty in hypothesis formulation/framing the research question/topic	17 (10.9%)	42 (26.9%)	28 (17.9%)	53 (34%)	16 (10.3%)
Lack of prior research on subject–poor literature support	19 (12.2%)	51 (32.7%)	29 (18.6%)	43 (27.6%)	14 (9%)
Difficulty in literature review	23 (14.7%)	53 (34%)	21 (13.5%)	46 (29.5%)	13 (8.3%)
Limited access to research articles due to paid access	17 (10.9%)	27 (17.3%)	10 (6.4%)	59 (37.8%)	43 (27.6%)
Faulty methodology	17 (10.9%)	34 (21.8%)	37 (23.7%)	51 (32.7%)	17 (10.9%)
Funding	12 (7.7%)	16 (10.3%)	22 (14.1%)	57 (36.5%)	49 (31.4%)
Data collection—lack of reliable data or representative data	15 (9.6%)	27 (17.3%)	24 (15.4%)	66 (42.3%)	24 (15.4%)
Dropouts/attrition	10 (6.4%)	24 (15.4%)	38 (24.4%)	64 (41%)	20 (12.8%)
Burnout	10 (6.4%)	20 (12.8%)	28 (17.9%)	73 (46.8%)	25 (16%)
Time constraints	5 (3.2%)	10 (6.4%)	13 (8.3%)	70 (44.9%)	58 (37.2%)
Lack of knowledge on statistical analysis	13 (8.3%)	18 (11.5%)	24 (15.4%)	57 (36.5%)	44 (28.2%)
Limited access to software for research analysis	11 (7.1%)	25 (16%)	26 (16.7%)	54 (34.6%)	40 (25.6%)
Lack of knowledge about formatting/documentation/editing/proofreading	16 (10.3%)	39 (25%)	20 (12.8%)	57 (36.5%)	24 (15.4%)
Difficulty in expression of language during writing up	23 (14.7%)	49 (31.4%)	26 (16.7%)	43 (27.6%)	15 (9.6%)
Lack of guidance/supervision	12 (7.7%)	37 (23.7%)	21 (13.5%)	56 (35.9%)	30 (19.2%)
Difficulty in finding related references	21 (13.5%)	50 (32.1%)	27 (17.3%)	39 (25%)	19 (12.2%)
Lack of interest in research	38 (24.4%)	42 (26.9%)	27 (17.3%)	37 (23.7%)	12 (7.7%)
Difficulty in staying motivated	19 (12.2%)	38 (24.4%)	30 (19.2%)	50 (32.1%)	19 (12.2%)
Writing introduction/discussion	22 (14.1%)	48 (30.8%)	37 (23.7%)	41 (26.3%)	8 (5.1%)
Absence of research partner	15 (9.6%)	28 (17.9%)	29 (18.6%)	54 (34.6%)	30 (19.2%)

**Table 4:** Limitations in scientific paper writing.

What do you think are the limitations in scientific paper writing? (*n* = 156)	Strongly disagree	Disagree	Neither agree/nor disagree	Agree	Strongly agree
I'm afraid my scientific paper may not receive good number of citations despite the hardwork	17 (10.9%)	30 (19.2%)	42 (26.9%)	53 (34%)	14 (9%)
I find it difficult to evade plagiarism and paraphrase	13 (8.3%)	41 (26.3%)	42 (26.9%)	50 (32.1%)	10 (6.4%)
I have financial constraints	8 (5.1%)	31 (19.9%)	30 (19.2%)	61 (39.1%)	26 (16.7%)
I find it difficult to seek ethics committee approval	14 (9%)	40 (25.6%)	40 (25.6%)	50 (32.1%)	12 (7.7%)
I have faced gender bias	21 (13.5%)	63 (40.4%)	47 (30.1%)	16 (10.3%)	9 (5.8%)
Article processing charges for journals are too high	8 (5.1%)	21 (13.5%)	17 (10.9%)	57 (36.5%)	53 (34%)
Demand for increased number of revisions by the editors puts me off	5 (3.2%)	30 (19.2%)	38 (24.4%)	63 (40.4%)	20 (12.8%)
I'm unable to keep up with the surge of artificial intelligence tools—ChatGPT	14 (9%)	38 (24.4%)	41 (26.3%)	48 (30.8%)	15 (9.6%)
I doubt if my results will have any major real-world impact	12 (7.7%)	35 (22.4%)	38 (24.4%)	55 (35.3%)	16 (10.3%)

## Data Availability

The data that support the findings of this study are available on request from the corresponding author. The data are not publicly available due to privacy or ethical restrictions.

## Supporting Information

Supporting Table 1: challenges in paper scripting in relation to age (in years) of doctors studied.

Supporting Table 2: challenges in paper scripting in relation to gender of doctors studied.
