# Supplementary material for: Barriers and Constraints in Scientific Manuscript Preparation Among Nephrologists: Insights From India
Source: Int J Nephrol. 2025 Feb 23;2025:9008616. doi: 10.1155/ijne/9008616 (PMC11872293; doi:10.1155/ijne/9008616)
Supplement: Supporting Information — Additional supporting information can be found online in the Supporting Information section. [file 9008616.f1.docx]

**Supplementary Table 1**: Challenges in paper scripting in relation to age in years of doctors studied

| **Variables** | **Age in Years** | | | | **Total** | **P Value** |
| --- | --- | --- | --- | --- | --- | --- |
|  | **21-30 yrs** | **31-40 yrs** | **41-50 yrs** | **51 yrs & more** |  |  |
| How many podium/poster presentations have you done so far? |  |  |  |  |  |  |
| - Less than 2 | 29(67.4%) | 19(22.6%) | 2(11.1%) | 1(9.1%) | 51(32.7%) | <0.001** |
| - 2-5 | 12(27.9%) | 39(46.4%) | 8(44.4%) | 1(9.1%) | 60(38.5%) |  |
| - More than 5 | 2(4.7%) | 26(31%) | 8(44.4%) | 9(81.8%) | 45(28.8%) |  |
| How many podium/poster presentations got converted into paper publications? |  |  |  |  |  |  |
| - Less than 2 | 41(95.3%) | 59(70.2%) | 11(61.1%) | 2(18.2%) | 113(72.4%) | <0.001** |
| - 2-5 | 1(2.3%) | 17(20.2%) | 2(11.1%) | 3(27.3%) | 23(14.7%) |  |
| - More than 5 | 1(2.3%) | 8(9.5%) | 5(27.8%) | 6(54.5%) | 20(12.8%) |  |
| Total | 43(100%) | 84(100%) | 18(100%) | 11(100%) | 156(100%) |  |
| **Difficulty in hypothesis formulation/ framing the research question/topic** |  |  |  |  |  |  |
| Strongly disagree | 3(7%) | 8(9.5%) | 5(27.8%) | 1(9.1%) | 17(10.9%) | 0.093+ |
| Disagree | 8(18.6%) | 27(32.1%) | 4(22.2%) | 3(27.3%) | 42(26.9%) |  |
| Neither agree /nor disagree | 9(20.9%) | 16(19%) | 1(5.6%) | 2(18.2%) | 28(17.9%) |  |
| Agree | 15(34.9%) | 29(34.5%) | 5(27.8%) | 4(36.4%) | 53(34%) |  |
| Strongly agree | 8(18.6%) | 4(4.8%) | 3(16.7%) | 1(9.1%) | 16(10.3%) |  |
| **Lack of prior research on subject - poor literature support** |  |  |  |  |  |  |
| Strongly disagree | 3(7%) | 10(11.9%) | 5(27.8%) | 1(9.1%) | 19(12.2%) | 0.178 |
| Disagree | 10(23.3%) | 30(35.7%) | 6(33.3%) | 5(45.5%) | 51(32.7%) |  |
| Neither agree /nor disagree | 7(16.3%) | 19(22.6%) | 3(16.7%) | 0(0%) | 29(18.6%) |  |
| Agree | 17(39.5%) | 20(23.8%) | 3(16.7%) | 3(27.3%) | 43(27.6%) |  |
| Strongly agree | 6(14%) | 5(6%) | 1(5.6%) | 2(18.2%) | 14(9%) |  |
| **Difficulty in literature review** |  |  |  |  |  |  |
| Strongly disagree | 5(11.6%) | 10(11.9%) | 6(33.3%) | 2(18.2%) | 23(14.7%) | 0.007** |
| Disagree | 7(16.3%) | 31(36.9%) | 7(38.9%) | 8(72.7%) | 53(34%) |  |
| Neither agree /nor disagree | 9(20.9%) | 12(14.3%) | 0(0%) | 0(0%) | 21(13.5%) |  |
| Agree | 17(39.5%) | 26(31%) | 3(16.7%) | 0(0%) | 46(29.5%) |  |
| Strongly agree | 5(11.6%) | 5(6%) | 2(11.1%) | 1(9.1%) | 13(8.3%) |  |
| **Limited access to research articles due to paid access** |  |  |  |  |  |  |
| Strongly disagree | 2(4.7%) | 10(11.9%) | 4(22.2%) | 1(9.1%) | 17(10.9%) | 0.130 |
| Disagree | 6(14%) | 16(19%) | 1(5.6%) | 4(36.4%) | 27(17.3%) |  |
| Neither agree /nor disagree | 1(2.3%) | 6(7.1%) | 2(11.1%) | 1(9.1%) | 10(6.4%) |  |
| Agree | 18(41.9%) | 29(34.5%) | 10(55.6%) | 2(18.2%) | 59(37.8%) |  |
| Strongly agree | 16(37.2%) | 23(27.4%) | 1(5.6%) | 3(27.3%) | 43(27.6%) |  |
| **Faulty methodology** |  |  |  |  |  |  |
|          Strongly disagree | 3(7%) | 8(9.5%) | 3(16.7%) | 3(27.3%) | 17(10.9%) | 0.631 |
|          Disagree | 6(14%) | 21(25%) | 4(22.2%) | 3(27.3%) | 34(21.8%) |  |
|          Neither agree /nor disagree | 14(32.6%) | 16(19%) | 5(27.8%) | 2(18.2%) | 37(23.7%) |  |
|          Agree | 15(34.9%) | 29(34.5%) | 5(27.8%) | 2(18.2%) | 51(32.7%) |  |
|          Strongly agree | 5(11.6%) | 10(11.9%) | 1(5.6%) | 1(9.1%) | 17(10.9%) |  |
| **Funding** |  |  |  |  |  |  |
|          Strongly disagree | 1(2.3%) | 8(9.5%) | 2(11.1%) | 1(9.1%) | 12(7.7%) | 0.010* |
|          Disagree | 5(11.6%) | 5(6%) | 2(11.1%) | 4(36.4%) | 16(10.3%) |  |
|          Neither agree /nor disagree | 5(11.6%) | 14(16.7%) | 3(16.7%) | 0(0%) | 22(14.1%) |  |
|          Agree | 24(55.8%) | 29(34.5%) | 3(16.7%) | 1(9.1%) | 57(36.5%) |  |
|          Strongly agree | 8(18.6%) | 28(33.3%) | 8(44.4%) | 5(45.5%) | 49(31.4%) |  |
| **Data collection- Lack of reliable data or representative data** |  |  |  |  |  |  |
|          Strongly disagree | 4(9.3%) | 8(9.5%) | 2(11.1%) | 1(9.1%) | 15(9.6%) | 0.997 |
|          Disagree | 6(14%) | 14(16.7%) | 4(22.2%) | 3(27.3%) | 27(17.3%) |  |
|          Neither agree /nor disagree | 8(18.6%) | 13(15.5%) | 2(11.1%) | 1(9.1%) | 24(15.4%) |  |
|          Agree | 19(44.2%) | 35(41.7%) | 8(44.4%) | 4(36.4%) | 66(42.3%) |  |
|          Strongly agree | 6(14%) | 14(16.7%) | 2(11.1%) | 2(18.2%) | 24(15.4%) |  |
| **Drop outs/ attrition** |  |  |  |  |  |  |
|          Strongly disagree | 3(7%) | 6(7.1%) | 1(5.6%) | 0(0%) | 10(6.4%) | 0.717 |
|          Disagree | 5(11.6%) | 12(14.3%) | 3(16.7%) | 4(36.4%) | 24(15.4%) |  |
|          Neither agree /nor disagree | 10(23.3%) | 21(25%) | 4(22.2%) | 3(27.3%) | 38(24.4%) |  |
|          Agree | 16(37.2%) | 36(42.9%) | 8(44.4%) | 4(36.4%) | 64(41%) |  |
|          Strongly agree | 9(20.9%) | 9(10.7%) | 2(11.1%) | 0(0%) | 20(12.8%) |  |
| **Burn out** |  |  |  |  |  |  |
|          Strongly disagree | 3(7%) | 6(7.1%) | 1(5.6%) | 0(0%) | 10(6.4%) | 0.002** |
|          Disagree | 1(2.3%) | 9(10.7%) | 5(27.8%) | 5(45.5%) | 20(12.8%) |  |
|          Neither agree /nor disagree | 7(16.3%) | 15(17.9%) | 3(16.7%) | 3(27.3%) | 28(17.9%) |  |
|          Agree | 24(55.8%) | 43(51.2%) | 5(27.8%) | 1(9.1%) | 73(46.8%) |  |
|          Strongly agree | 8(18.6%) | 11(13.1%) | 4(22.2%) | 2(18.2%) | 25(16%) |  |
| **Time constraints** |  |  |  |  |  |  |
|          Strongly disagree | 1(2.3%) | 3(3.6%) | 1(5.6%) | 0(0%) | 5(3.2%) | 0.600 |
|          Disagree | 2(4.7%) | 6(7.1%) | 1(5.6%) | 1(9.1%) | 10(6.4%) |  |
|          Neither agree /nor disagree | 4(9.3%) | 4(4.8%) | 2(11.1%) | 3(27.3%) | 13(8.3%) |  |
|          Agree | 22(51.2%) | 38(45.2%) | 7(38.9%) | 3(27.3%) | 70(44.9%) |  |
|          Strongly agree | 14(32.6%) | 33(39.3%) | 7(38.9%) | 4(36.4%) | 58(37.2%) |  |
| **Lack of knowledge on statistical analysis** |  |  |  |  |  |  |
|          Strongly disagree | 3(7%) | 8(9.5%) | 2(11.1%) | 0(0%) | 13(8.3%) | 0.012* |
|          Disagree | 1(2.3%) | 10(11.9%) | 5(27.8%) | 2(18.2%) | 18(11.5%) |  |
|          Neither agree /nor disagree | 7(16.3%) | 9(10.7%) | 6(33.3%) | 2(18.2%) | 24(15.4%) |  |
|          Agree | 21(48.8%) | 29(34.5%) | 2(11.1%) | 5(45.5%) | 57(36.5%) |  |
|          Strongly agree | 11(25.6%) | 28(33.3%) | 3(16.7%) | 2(18.2%) | 44(28.2%) |  |
| **Limited access to software for research analysis** |  |  |  |  |  |  |
|          Strongly disagree | 1(2.3%) | 7(8.3%) | 3(16.7%) | 0(0%) | 11(7.1%) | 0.040* |
|          Disagree | 3(7%) | 13(15.5%) | 6(33.3%) | 3(27.3%) | 25(16%) |  |
|          Neither agree /nor disagree | 11(25.6%) | 10(11.9%) | 3(16.7%) | 2(18.2%) | 26(16.7%) |  |
|          Agree | 20(46.5%) | 26(31%) | 4(22.2%) | 4(36.4%) | 54(34.6%) |  |
|          Strongly agree | 8(18.6%) | 28(33.3%) | 2(11.1%) | 2(18.2%) | 40(25.6%) |  |
| **Lack of knowledge about formatting/documentation/editing/ proofreading** |  |  |  |  |  |  |
|          Strongly disagree | 3(7%) | 9(10.7%) | 3(16.7%) | 1(9.1%) | 16(10.3%) | 0.248 |
|          Disagree | 6(14%) | 24(28.6%) | 7(38.9%) | 2(18.2%) | 39(25%) |  |
|          Neither agree /nor disagree | 7(16.3%) | 8(9.5%) | 4(22.2%) | 1(9.1%) | 20(12.8%) |  |
|          Agree | 21(48.8%) | 28(33.3%) | 2(11.1%) | 6(54.5%) | 57(36.5%) |  |
|          Strongly agree | 6(14%) | 15(17.9%) | 2(11.1%) | 1(9.1%) | 24(15.4%) |  |
| **Difficulty in expression of language during writing up** |  |  |  |  |  |  |
|          Strongly disagree | 5(11.6%) | 11(13.1%) | 4(22.2%) | 3(27.3%) | 23(14.7%) | 0.256 |
|          Disagree | 8(18.6%) | 30(35.7%) | 6(33.3%) | 5(45.5%) | 49(31.4%) |  |
|          Neither agree /nor disagree | 12(27.9%) | 10(11.9%) | 3(16.7%) | 1(9.1%) | 26(16.7%) |  |
|          Agree | 12(27.9%) | 27(32.1%) | 3(16.7%) | 1(9.1%) | 43(27.6%) |  |
|          Strongly agree | 6(14%) | 6(7.1%) | 2(11.1%) | 1(9.1%) | 15(9.6%) |  |
| **Lack of guidance/ supervision** |  |  |  |  |  |  |
|          Strongly disagree | 3(7%) | 5(6%) | 3(16.7%) | 1(9.1%) | 12(7.7%) | 0.688 |
|          Disagree | 5(11.6%) | 24(28.6%) | 4(22.2%) | 4(36.4%) | 37(23.7%) |  |
|          Neither agree /nor disagree | 7(16.3%) | 10(11.9%) | 2(11.1%) | 2(18.2%) | 21(13.5%) |  |
|          Agree | 19(44.2%) | 29(34.5%) | 6(33.3%) | 2(18.2%) | 56(35.9%) |  |
|          Strongly agree | 9(20.9%) | 16(19%) | 3(16.7%) | 2(18.2%) | 30(19.2%) |  |
| **Difficulty in finding related references** |  |  |  |  |  |  |
|          Strongly disagree | 2(4.7%) | 13(15.5%) | 4(22.2%) | 2(18.2%) | 21(13.5%) | 0.122 |
|          Disagree | 11(25.6%) | 26(31%) | 9(50%) | 4(36.4%) | 50(32.1%) |  |
|          Neither agree /nor disagree | 6(14%) | 19(22.6%) | 1(5.6%) | 1(9.1%) | 27(17.3%) |  |
|          Agree | 18(41.9%) | 16(19%) | 3(16.7%) | 2(18.2%) | 39(25%) |  |
|          Strongly agree | 6(14%) | 10(11.9%) | 1(5.6%) | 2(18.2%) | 19(12.2%) |  |
| **Lack of interest in research** |  |  |  |  |  |  |
|          Strongly disagree | 5(11.6%) | 22(26.2%) | 8(44.4%) | 3(27.3%) | 38(24.4%) | 0.1333 |
|          Disagree | 13(30.2%) | 23(27.4%) | 3(16.7%) | 3(27.3%) | 42(26.9%) |  |
|          Neither agree /nor disagree | 9(20.9%) | 13(15.5%) | 4(22.2%) | 1(9.1%) | 27(17.3%) |  |
|          Agree | 12(27.9%) | 22(26.2%) | 2(11.1%) | 1(9.1%) | 37(23.7%) |  |
|          Strongly agree | 4(9.3%) | 4(4.8%) | 1(5.6%) | 3(27.3%) | 12(7.7%) |  |
| **Difficulty in staying motivated** |  |  |  |  |  |  |
|          Strongly disagree | 4(9.3%) | 9(10.7%) | 4(22.2%) | 2(18.2%) | 19(12.2%) | 0.359 |
|          Disagree | 9(20.9%) | 21(25%) | 6(33.3%) | 2(18.2%) | 38(24.4%) |  |
|          Neither agree /nor disagree | 8(18.6%) | 18(21.4%) | 3(16.7%) | 1(9.1%) | 30(19.2%) |  |
|          Agree | 18(41.9%) | 27(32.1%) | 3(16.7%) | 2(18.2%) | 50(32.1%) |  |
|          Strongly agree | 4(9.3%) | 9(10.7%) | 2(11.1%) | 4(36.4%) | 19(12.2%) |  |
| **Writing introduction/ discussion** |  |  |  |  |  |  |
|          Strongly disagree | 3(7%) | 14(16.7%) | 3(16.7%) | 2(18.2%) | 22(14.1%) | 0.654 |
|          Disagree | 9(20.9%) | 28(33.3%) | 7(38.9%) | 4(36.4%) | 48(30.8%) |  |
|          Neither agree /nor disagree | 13(30.2%) | 17(20.2%) | 4(22.2%) | 3(27.3%) | 37(23.7%) |  |
|          Agree | 15(34.9%) | 22(26.2%) | 3(16.7%) | 1(9.1%) | 41(26.3%) |  |
|          Strongly agree | 3(7%) | 3(3.6%) | 1(5.6%) | 1(9.1%) | 8(5.1%) |  |
| **Absence of research Partner** |  |  |  |  |  |  |
|          Strongly disagree | 3(7%) | 10(11.9%) | 2(11.1%) | 0(0%) | 15(9.6%) | 0.303 |
|          Disagree | 4(9.3%) | 14(16.7%) | 6(33.3%) | 4(36.4%) | 28(17.9%) |  |
|          Neither agree /nor disagree | 9(20.9%) | 16(19%) | 3(16.7%) | 1(9.1%) | 29(18.6%) |  |
|          Agree | 18(41.9%) | 28(33.3%) | 6(33.3%) | 2(18.2%) | 54(34.6%) |  |
|          Strongly agree | 9(20.9%) | 16(19%) | 1(5.6%) | 4(36.4%) | 30(19.2%) |  |
| Total | 43(100%) | 84(100%) | 18(100%) | 11(100%) | 156(100%) |  |
| **On an average, how many journals rejected your paper before being accepted by one journal?** |  |  |  |  |  |  |
| Less than 2 | 17(39.5%) | 35(41.7%) | 4(22.2%) | 6(54.5%) | 62(39.7%) | 0.035* |
| 2-5 | 5(11.6%) | 29(34.5%) | 8(44.4%) | 4(36.4%) | 46(29.5%) |  |
| >5 | 1(2.3%) | 1(1.2%) | 1(5.6%) | 0(0%) | 3(1.9%) |  |
| Not applicable | 20(46.5%) | 19(22.6%) | 5(27.8%) | 1(9.1%) | 45(28.8%) |  |
| **On an average, after how many revisions were your papers accepted?** |  |  |  |  |  |  |
| Less than 2 | 13(30.2%) | 31(36.9%) | 5(27.8%) | 5(45.5%) | 54(34.6%) | 0.011* |
| 2-5 | 8(18.6%) | 37(44%) | 9(50%) | 5(45.5%) | 59(37.8%) |  |
| More than 5 | 0(0%) | 1(1.2%) | 0(0%) | 0(0%) | 1(0.6%) |  |
| Not Applicable | 22(51.2%) | 15(17.9%) | 4(22.2%) | 1(9.1%) | 42(26.9%) |  |
| Total | 43(100%) | 84(100%) | 18(100%) | 11(100%) | 156(100%) |  |

Supplementary table 2: Challenges in paper scripting in relation to gender of doctors studied

| **Variables** | **Gender** | | **Total** | **P value** |
| --- | --- | --- | --- | --- |
|  | **Female** | **Male** |  |  |
| How many podium/poster presentations have you done so far? |  |  |  |  |
| - Less than 2 | 22(31.9%) | 29(33.3%) | 51(32.7%) | 0.008** |
| - 2-5 | 19(27.5%) | 41(47.1%) | 60(38.5%) |  |
| - More than 5 | 28(40.6%) | 17(19.5%) | 45(28.8%) |  |
| How many podium/poster presentations got converted into paper publications? |  |  |  |  |
| - Less than 2 | 43(62.3%) | 70(80.5%) | 113(72.4%) | 0.036* |
| - 2-5 | 13(18.8%) | 10(11.5%) | 23(14.7%) |  |
| - More than 5 | 13(18.8%) | 7(8%) | 20(12.8%) |  |
| Total | 69(100%) | 87(100%) | 156(100%) |  |
| **Difficulty in hypothesis formulation/ framing the research question/topic** |  |  |  |  |
|          Strongly disagree | 11(15.9%) | 6(6.9%) | 17(10.9%) | 0.026* |
|          Disagree | 22(31.9%) | 20(23%) | 42(26.9%) |  |
|          Neither agree /nor disagree | 9(13%) | 19(21.8%) | 28(17.9%) |  |
|          Agree | 17(24.6%) | 36(41.4%) | 53(34%) |  |
|          Strongly agree | 10(14.5%) | 6(6.9%) | 16(10.3%) |  |
| **Lack of prior research on subject - Poor literature support** |  |  |  |  |
|          Strongly disagree | 10(14.5%) | 9(10.3%) | 19(12.2%) | 0.052+ |
|          Disagree | 27(39.1%) | 24(27.6%) | 51(32.7%) |  |
|          Neither agree /nor disagree | 6(8.7%) | 23(26.4%) | 29(18.6%) |  |
|          Agree | 21(30.4%) | 22(25.3%) | 43(27.6%) |  |
|          Strongly agree | 5(7.2%) | 9(10.3%) | 14(9%) |  |
| **Difficulty in literature review** |  |  |  |  |
|          Strongly disagree | 11(15.9%) | 12(13.8%) | 23(14.7%) | 0.945 |
|          Disagree | 24(34.8%) | 29(33.3%) | 53(34%) |  |
|          Neither agree /nor disagree | 10(14.5%) | 11(12.6%) | 21(13.5%) |  |
|          Agree | 18(26.1%) | 28(32.2%) | 46(29.5%) |  |
|          Strongly agree | 6(8.7%) | 7(8%) | 13(8.3%) |  |
| **Limited access to research articles due to paid access** |  |  |  |  |
|          Strongly disagree | 9(13%) | 8(9.2%) | 17(10.9%) | 0.114 |
|          Disagree | 9(13%) | 18(20.7%) | 27(17.3%) |  |
|          Neither agree /nor disagree | 3(4.3%) | 7(8%) | 10(6.4%) |  |
|          Agree | 33(47.8%) | 26(29.9%) | 59(37.8%) |  |
|          Strongly agree | 15(21.7%) | 28(32.2%) | 43(27.6%) |  |
| **Faulty methodology** |  |  |  |  |
|          Strongly disagree | 9(13%) | 8(9.2%) | 17(10.9%) | 0.535 |
|          Disagree | 15(21.7%) | 19(21.8%) | 34(21.8%) |  |
|          Neither agree /nor disagree | 12(17.4%) | 25(28.7%) | 37(23.7%) |  |
|          Agree | 25(36.2%) | 26(29.9%) | 51(32.7%) |  |
|          Strongly agree | 8(11.6%) | 9(10.3%) | 17(10.9%) |  |
| **Funding** |  |  |  |  |
|          Strongly disagree | 7(10.1%) | 5(5.7%) | 12(7.7%) | 0.546 |
|          Disagree | 8(11.6%) | 8(9.2%) | 16(10.3%) |  |
|          Neither agree /nor disagree | 12(17.4%) | 10(11.5%) | 22(14.1%) |  |
|          Agree | 23(33.3%) | 34(39.1%) | 57(36.5%) |  |
|          Strongly agree | 19(27.5%) | 30(34.5%) | 49(31.4%) |  |
| **Data collection- Lack of reliable data or representative data** |  |  |  |  |
|          Strongly disagree | 8(11.6%) | 7(8%) | 15(9.6%) | 0.567 |
|          Disagree | 13(18.8%) | 14(16.1%) | 27(17.3%) |  |
|          Neither agree /nor disagree | 7(10.1%) | 17(19.5%) | 24(15.4%) |  |
|          Agree | 30(43.5%) | 36(41.4%) | 66(42.3%) |  |
|          Strongly agree | 11(15.9%) | 13(14.9%) | 24(15.4%) |  |
| **Drop outs/ Attrition** |  |  |  |  |
|          Strongly disagree | 5(7.2%) | 5(5.7%) | 10(6.4%) | 0.962 |
|          Disagree | 11(15.9%) | 13(14.9%) | 24(15.4%) |  |
|          Neither agree /nor disagree | 16(23.2%) | 22(25.3%) | 38(24.4%) |  |
|          Agree | 27(39.1%) | 37(42.5%) | 64(41%) |  |
|          Strongly agree | 10(14.5%) | 10(11.5%) | 20(12.8%) |  |
| **Burn out** |  |  |  |  |
|          Strongly disagree | 4(5.8%) | 6(6.9%) | 10(6.4%) | 0.911 |
|          Disagree | 10(14.5%) | 10(11.5%) | 20(12.8%) |  |
|          Neither agree /nor disagree | 14(20.3%) | 14(16.1%) | 28(17.9%) |  |
|          Agree | 31(44.9%) | 42(48.3%) | 73(46.8%) |  |
|          Strongly agree | 10(14.5%) | 15(17.2%) | 25(16%) |  |
| **Time constraints** |  |  |  |  |
|          Strongly disagree | 2(2.9%) | 3(3.4%) | 5(3.2%) | 0.946 |
|          Disagree | 5(7.2%) | 5(5.7%) | 10(6.4%) |  |
|          Neither agree /nor disagree | 7(10.1%) | 6(6.9%) | 13(8.3%) |  |
|          Agree | 30(43.5%) | 40(46%) | 70(44.9%) |  |
|          Strongly agree | 25(36.2%) | 33(37.9%) | 58(37.2%) |  |
| **Lack of knowledge on statistical analysis** |  |  |  |  |
|          Strongly disagree | 6(8.6%) | 7(8%) | 13(8.3%) | 0.702 |
|          Disagree | 10(14.5%) | 8(9.2%) | 18(11.5%) |  |
|          Neither agree /nor disagree | 9(13%) | 15(17.2%) | 24(15.4%) |  |
|          Agree | 27(39.1%) | 30(34.5%) | 57(36.5%) |  |
|          Strongly agree | 17(24.6%) | 27(31%) | 44(28.2%) |  |
| **Limited access to software for research analysis** |  |  |  |  |
|          Strongly disagree | 6(8.7%) | 5(5.7%) | 11(7.1%) | 0.731 |
|          Disagree | 10(14.5%) | 15(17.2%) | 25(16%) |  |
|          Neither agree /nor disagree | 10(14.5%) | 16(18.4%) | 26(16.7%) |  |
|          Agree | 27(39.1%) | 27(31%) | 54(34.6%) |  |
|          Strongly agree | 16(23.2%) | 24(27.6%) | 40(25.6%) |  |
| **Lack of knowledge about formatting/documentation/editing/ proofreading** |  |  |  |  |
|          Strongly disagree | 6(8.7%) | 10(11.5%) | 16(10.3%) | 0.673 |
|          Disagree | 21(30.4%) | 18(20.7%) | 39(25%) |  |
|          Neither agree /nor disagree | 8(11.6%) | 12(13.8%) | 20(12.8%) |  |
|          Agree | 25(36.2%) | 32(36.8%) | 57(36.5%) |  |
|          Strongly agree | 9(13%) | 15(17.2%) | 24(15.4%) |  |
| **Difficulty in expression of language during writing up** |  |  |  |  |
|          Strongly disagree | 14(20.3%) | 9(10.3%) | 23(14.7%) | 0.380 |
|          Disagree | 23(33.3%) | 26(29.9%) | 49(31.4%) |  |
|          Neither agree /nor disagree | 9(13%) | 17(19.5%) | 26(16.7%) |  |
|          Agree | 17(24.6%) | 26(29.9%) | 43(27.6%) |  |
|          Strongly agree | 6(8.7%) | 9(10.3%) | 15(9.6%) |  |
| **Lack of guidance/ supervision** |  |  |  |  |
|          Strongly disagree | 8(11.6%) | 4(4.6%) | 12(7.7%) | 0.274 |
|          Disagree | 16(23.2%) | 21(24.1%) | 37(23.7%) |  |
|          Neither agree /nor disagree | 12(17.4%) | 9(10.3%) | 21(13.5%) |  |
|          Agree | 21(30.4%) | 35(40.2%) | 56(35.9%) |  |
|          Strongly agree | 12(17.4%) | 18(20.7%) | 30(19.2%) |  |
| **Difficulty in finding related references** |  |  |  |  |
|          Strongly disagree | 12(17.4%) | 9(10.3%) | 21(13.5%) | 0.214 |
|          Disagree | 25(36.2%) | 25(28.7%) | 50(32.1%) |  |
|          Neither agree /nor disagree | 7(10.1%) | 20(23%) | 27(17.3%) |  |
|          Agree | 17(24.6%) | 22(25.3%) | 39(25%) |  |
|          Strongly agree | 8(11.6%) | 11(12.6%) | 19(12.2%) |  |
| **Lack of interest in research** |  |  |  |  |
|          Strongly disagree | 24(34.8%) | 14(16.1%) | 38(24.4%) | 0.018* |
|          Disagree | 21(30.4%) | 21(24.1%) | 42(26.9%) |  |
|          Neither agree /nor disagree | 7(10.1%) | 20(23%) | 27(17.3%) |  |
|          Agree | 12(17.4%) | 25(28.7%) | 37(23.7%) |  |
|          Strongly agree | 5(7.2%) | 7(8%) | 12(7.7%) |  |
| **Difficulty in staying motivated** |  |  |  |  |
|          Strongly disagree | 10(14.5%) | 9(10.3%) | 19(12.2%) | 0.092+ |
|          Disagree | 23(33.3%) | 15(17.2%) | 38(24.4%) |  |
|          Neither agree /nor disagree | 13(18.8%) | 17(19.5%) | 30(19.2%) |  |
|          Agree | 17(24.6%) | 33(37.9%) | 50(32.1%) |  |
|          Strongly agree | 6(8.7%) | 13(14.9%) | 19(12.2%) |  |
| **Writing introduction/ discussion** |  |  |  |  |
|          Strongly disagree | 12(17.4%) | 10(11.5%) | 22(14.1%) | 0.458 |
|          Disagree | 20(29%) | 28(32.2%) | 48(30.8%) |  |
|          Neither agree /nor disagree | 19(27.5%) | 18(20.7%) | 37(23.7%) |  |
|          Agree | 14(20.3%) | 27(31%) | 41(26.3%) |  |
|          Strongly agree | 4(5.8%) | 4(4.6%) | 8(5.1%) |  |
| **Absence of research partner** |  |  |  |  |
|          Strongly disagree | 7(10.1%) | 8(9.2%) | 15(9.6%) | 0.707 |
|          Disagree | 15(21.7%) | 13(14.9%) | 28(17.9%) |  |
|          Neither agree /nor disagree | 10(14.5%) | 19(21.8%) | 29(18.6%) |  |
|          Agree | 24(34.8%) | 30(34.5%) | 54(34.6%) |  |
|          Strongly agree | 13(18.8%) | 17(19.5%) | 30(19.2%) |  |
| Total | 69(100%) | 87(100%) | 156(100%) |  |
| **On an average, how many times were your papers rejected before being accepted?** |  |  |  |  |
| Less than 2 | 26(37.7%) | 36(41.4%) | 62(39.7%) | 0.877 |
| 2-5 | 20(29%) | 26(29.9%) | 46(29.5%) |  |
| More than 5 | 1(1.4%) | 2(2.3%) | 3(1.9%) |  |
| Not Applicable | 22(31.9%) | 23(26.4%) | 45(28.8%) |  |
| **On an average, after how many revisions were your papers accepted?** |  |  |  |  |
| Less than 2 | 23(33.3%) | 31(35.6%) | 54(34.6%) | 0.790 |
| 2-5 | 26(37.7%) | 33(37.9%) | 59(37.8%) |  |
| More than 5 | 0(0%) | 1(1.1%) | 1(0.6%) |  |
| Not Applicable | 20(29%) | 22(25.3%) | 42(26.9%) |  |
| Total | 69(100%) | 87(100%) | 156(100%) |  |
